# Genome-wide association study (GWAS) of leaf wax components of apple

**DOI:** 10.1007/s44154-021-00012-3

**Published:** 2021-11-18

**Authors:** Fuguo Cao, Zhongxing Li, Lijuan Jiang, Chen Liu, Qian Qian, Feng Yang, Fengwang Ma, Qingmei Guan

**Affiliations:** Key Laboratory of Crop Stress Biology for Arid Areas/Shaanxi Key Laboratory of Apple, College of Horticulture, Northwest A&F University, Yangling, 712100 People’s Republic of China

**Keywords:** Apple, Leaves, Genome-wide association study (GWAS), Wax

## Abstract

The wax layer of apple leaves plays an important role in improving stress resistance, but relatively little is known about the mechanisms of wax synthesis and transport in apple leaves. In this study, 17 wax components, including alcohols, alkanes, fatty acids and terpenes, were analyzed by gas chromatography-tandem mass spectrometry (GC-MS) from the leaves of 123 apple germplasms. Whole-genome sequencing of these apple accessions yielded 5.9 million high-quality single nucleotide polymorphisms (SNPs). We performed a genome-wide association study (GWAS) on 17 wax components and identified several genes related to wax synthesis and transport, including *MdSHN1* (*SHINE1)*, *MdLTP4 (LIPID TRANSFER PROTEIN4)*, *MdWSD1* (*WAX ESTER SYNTHASE/ACYL-COA DIAC-YLGLYCEROL ACYLTRANSFERASE1)*, *MdRDR1 (RNA-DEPENDENT RNA POLYMERASE1)*, *MdACBP6 (ACYL-COA-BINDING PROTEIN6)*, *MdNLE (NOTCHLESS)* and *MdABCG21* (*ATP-BINDING CASSETTE G21)*. Moreover, we identified some prominent SNPs that may affect gene expression and protein function. These results provide insights into mechanisms of wax synthesis and transport in apple leaves and broaden the genetic resources and basis for facilitating resistance breeding.

**Supplementary Information:**

The online version contains supplementary material available at 10.1007/s44154-021-00012-3.

## Introduction

Apples contain a wide range of antioxidants and have an overall high nutritional value, making them beneficial for human health (Boyer and Liu 2004). Global apple production in 2018 exceeded 86 million tons (FAOSTAT, http://www.fao.org/faostat/). However, biotic and abiotic stressors that commonly exist in apple production regions often severely impact the yield and quality of apples (Mittelberger et al. 2017; Song et al. 2019).

In order to deal with these stressors, a layer of lipid, called the cuticle, covers the epidermis of the aboveground parts of plants. The cuticle is secreted by epidermal cells and is widely found in stems, leaves, flowers, fruits and other tissues (Buschhaus and Jetter 2011; Yeats and Rose 2013). Wax is an important component of the plant cuticle. According to previous reports, the wax of leaves improves plant drought resistance by preventing the loss of non-stomatal water and plays an important role in cold resistance (Kerstiens 2006; Kunst and Samuels 2003; Liao et al. 2014). In addition, Wax improves plant disease resistance by preventing direct contact with bacteria (Solovchenko and Merzlyak 2003; Yeats and Rose 2013). In plant reproductive organs, wax also plays an important role in preventing genital fusion (Aarts et al. 1995).

Especially in the arid regions in Northwest China, the most important factor affecting plant growth is drought stress, which can lead to decreases of crop yields (Zhang et al. 2015). Rice and wheat plants with higher wax content have stronger drought tolerance and higher yields under drought stress than those with lower wax content (Guo et al. 2016; Zhou et al. 2013). The composition of the wax itself can also impact drought resistance. Alkanes account for 93% of the plant cuticle wax under drought, and increases of wax that occur under drought are mainly due to increases of long-chain alkanes, especially C29, C31 and C33 (Kosma et al. 2009). Similarly, previous studies have shown that a decrease of C29 alkane in cuticle wax reduces drought tolerance (Panikashvili et al. 2007).

Wax is usually composed of very long chain fatty acids (VLCFAs) and their derivatives, including primary alcohols, secondary alcohols, ketones, aldehydes, esters and alkanes (Li-Beisson et al. 2013; Pollard et al. 2008; Samuels et al. 2008). A complex series of enzymatic processes leads to the synthesis of the very-long-chain fatty acids (VLCFAs) that constitute plant epidermal wax. In plastids, malonyl-acyl carrier protein (malonyl-ACP) is a donor of carbon atoms for chain elongation, and the fatty acid synthase (FAS) complex catalyzes the combinations of acetyl-CoA and malonyl-ACP into C16 and C18 long-chain fatty acids, including palmitic acid (16:0), stearic acid (18:0) and oleic acid (18:1) (Li-Beisson et al. 2013; Shepherd and Wynne Griffiths 2006; Troncoso-Ponce et al. 2016; Wang and Benning 2012). The resulting C16 and C18 acyl ACPs are utilized in plastids by acyl ACP thiolase to form the corresponding fatty acid, which is used by long-chain acyl coenzyme A synthetase (LACS) to form acyl CoA (Dormann et al. 1995; Jones et al. 1995; Lu et al. 2009).

Acyl CoA is subsequently transported to the endoplasmic reticulum where it acts as a receptor for further chain extension. Acetyl CoA is utilized by acetyl-CoA carboxylase (ACC) 1 to form malonyl CoA, which is the carbon donor for the chain extension (Lu et al. 2011). The carbon chain is extended under the catalysis of the fatty acid elongase (FAE) complex located in the endoplasmic reticulum. FAE contains four enzymes, *β*-ketoacyl-CoA synthase (KCS), *β*-ketoacyl-CoA reductase (KCR), *β*-hydroxyacyl-CoA dehydratase (HCD) and enoyl-CoA reductase (ECR) (Bach et al. 2008; Beaudoin et al. 2009; Kim et al. 2013; Zheng et al. 2005). These four reactions constitute a cycle in the extension process of VLCFAs, in which each cycle adds two carbon atoms. A particular fatty acid chain undergoes many cycles until it achieves the ideal carbon chain length, as each the fatty acid in wax is generally composed of between 20 and 34 carbon units (Kunst and Samuels 2009).

In addition to the production of VLCFAs, waxes on the epidermis are mainly synthesized by the alcohol synthesis and alkane synthesis pathways (Kunst and Samuels 2009; Samuels et al. 2008). Primary alcohols and wax esters are produced by the alcohol synthesis pathway, with the *WSD1* (a member of the bifunctional wax ester synthase/diacylglycerol acyltransferase gene family) having been shown to play a key role in the synthesis of wax esters in the stem of *Arabidopsis thaliana* (Li et al. 2008). Alkanes, secondary alcohols, aldehydes and ketones are produced by the alkane biosynthesis pathway. Here, *ECERIFERUM 1* (*CER1)* and *ECERIFERUM 3* (*CER3)* are known to be particularly important genes involved in the pathway of alkane synthesis in Arabidopsis (Bourdenx et al. 2011; Rowland et al. 2007).

Various components of wax are synthesized in the endoplasmic reticulum. Therefore, the waxes must pass through the plasma membrane to the apoplast and through the cell wall to reach the plant epidermis (Kunst and Samuels 2003). Previous studies have shown that acyl-CoA binding proteins (ACBPs) have a conserved acyl CoA binding (ACB) domain, which can bind to long-chain fatty acyl CoA. In Arabidopsis, *acbp1* mutants had lower stem wax content than the wild type, suggesting that *AtACBP1* is involved in the formation of the Arabidopsis stem epidermis through the transport of lipoyl CoA (Xue et al. 2014). *ATP-BINDING CASSETTE G12 (ABCG12)* and *ATP-BINDING CASSETTE G11 (ABCG11)*, which encode two ATP-binding cassette (ABC) transporters, have also been shown to be involved in wax transport (Bird et al. 2007; Kunst and Samuels 2009; Pighin et al. 2004). Other studies have also shown that the genes encoding lipid transfer proteins (LTPs) are up-regulated during drought stress and that they induce cuticular wax accumulation, indicating that these proteins are also important to wax accumulation (Cameron et al. 2006).

The recent identification of these and other genes involved in wax synthesis is important, because wax synthesis is mainly controlled by regulation at the transcriptional and post-translational levels. Elucidation of the relevant genes supports the current focus on understanding transcriptional regulation. For example, several critical transcription factors, including members of the APETALA2 (AP2)/ethylene-responsive element binding factor (ERF/EREBP), MYB and homeodomain–leucine zipper (HD-ZIP) IV families have been found to regulate wax biosynthesis. In particular, with regard to the AP2 family, *WAX INDUCER1/SHINE1* (*WIN1/SHN1)*, *WXP1*, *DECREASE WAX BIOSYNTHESIS (DEWAX)* have been found to regulate wax biosynthesis (Aharoni et al. 2004; Go et al. 2014; Kannangara et al. 2007; Zhang et al. 2007). MYB106 and MYB16 can cooperate with WIN1/SHN1 to regulate wax synthesis (Oshima et al. 2013). In Arabidopsis and rice, CURLY FLAG LEAF1 (CFL1) interacts with HOMEODOMAIN GLABRA1 (HDG1) negatively regulates wax biosynthesis (Wu et al. 2011). Post translational regulation of proteins involved in wax synthesis has also been reported. For example, *Oryza sativa* DROUGHT HYPERSENSITIVE (OsDHS) has E3 ubiquitin ligase activity and promotes the degradation of the ROC4 (an HD-ZIP IV family member) via the 26S proteasome to negatively regulate the biosynthesis of wax and affect the drought resistance of rice (Wang et al. 2018). MIEL1 affects wax synthesis in Arabidopsis by activating the 26S proteasomal degradation pathway of MYB96 and MYB30 (Lee and Seo 2016; Marino et al. 2013). *ECERIFERUM9 (CER9)* gene in Arabidopsis encodes an E3 ubiquitin ligase, which regulates early steps of wax synthesis (Lu et al. 2012).

Thus, many enzyme and regulatory networks have been identified in epidermal wax synthesis pathways. However, few studies have focused on the comprehensive characterization of potential genes impacting apple cuticular wax biosynthesis. Here, we applied genome-wide association study (GWAS) analysis in a large sample of apples (123 accessions) to study the genetic basis for the diversity of leaf wax content through robust analysis of genotype-phenotype association. Our results demonstrated several new candidate chromosomal sites that may serve as the focus of future research on wax synthesis. This study provides a basis for molecular breeding to improve drought resistance of apples by improving wax accumulation and reducing water loss under drought conditions.

## Results

### Wax content of apple accessions

**Fig. 1:**
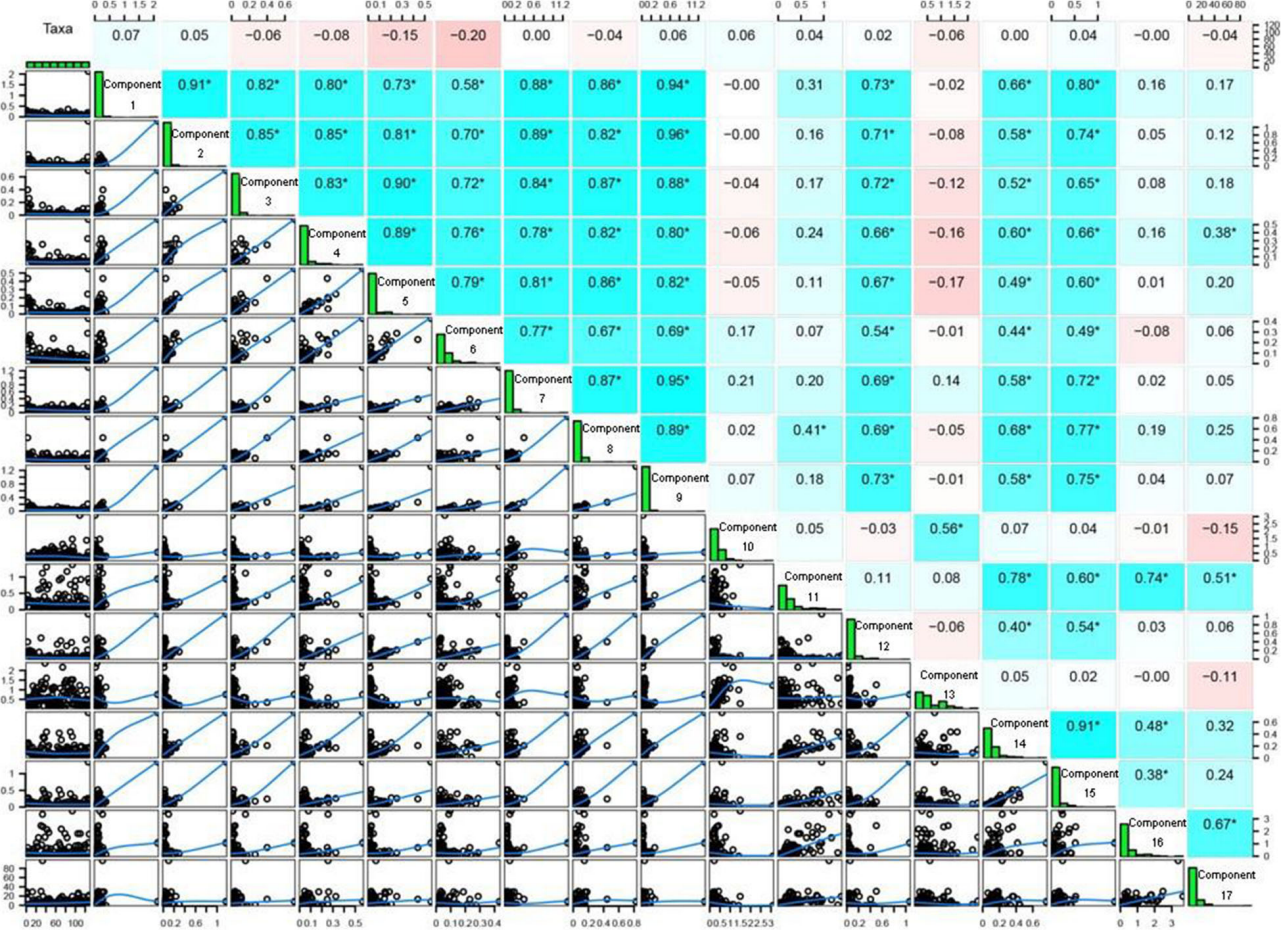
Pearson correlation analysis of wax components. Statistical analysis of leaf wax components. The upper half of the rectangles represents the correlation and significance between the components (*, *p* < 0.05; **, *p* < 0.01; ***, *p* < 0.001; the value is the *r*^2^ of the correlation analysis). The diagonal line represents the frequency distribution of the content of each of the wax components, and the bottom half of the rectangles represents the correlation between the two leaf wax components. The blue line is the fitting curve. (Component 1: Palmitic acid; Component 2: Linoleic acid; Component 3: Oleic acid; Component 4: Stearic acid; Component 5: Tricosane; Component 6: Pentacosane; Component 7: Heptacosane; Component 8: Tetracosanol; Component 9: Lignoceric acid; Component 10: Nonacosane; Component 11: Hexacosanol; Component 12: Hexacosanoic acid; Component 13: Hentriacontane; Component 14: Octacosanol; Component 15: Triacontanol; Component 16: Oleanolic acid; Component 17: Ursolic acid)

**Fig. 2:**
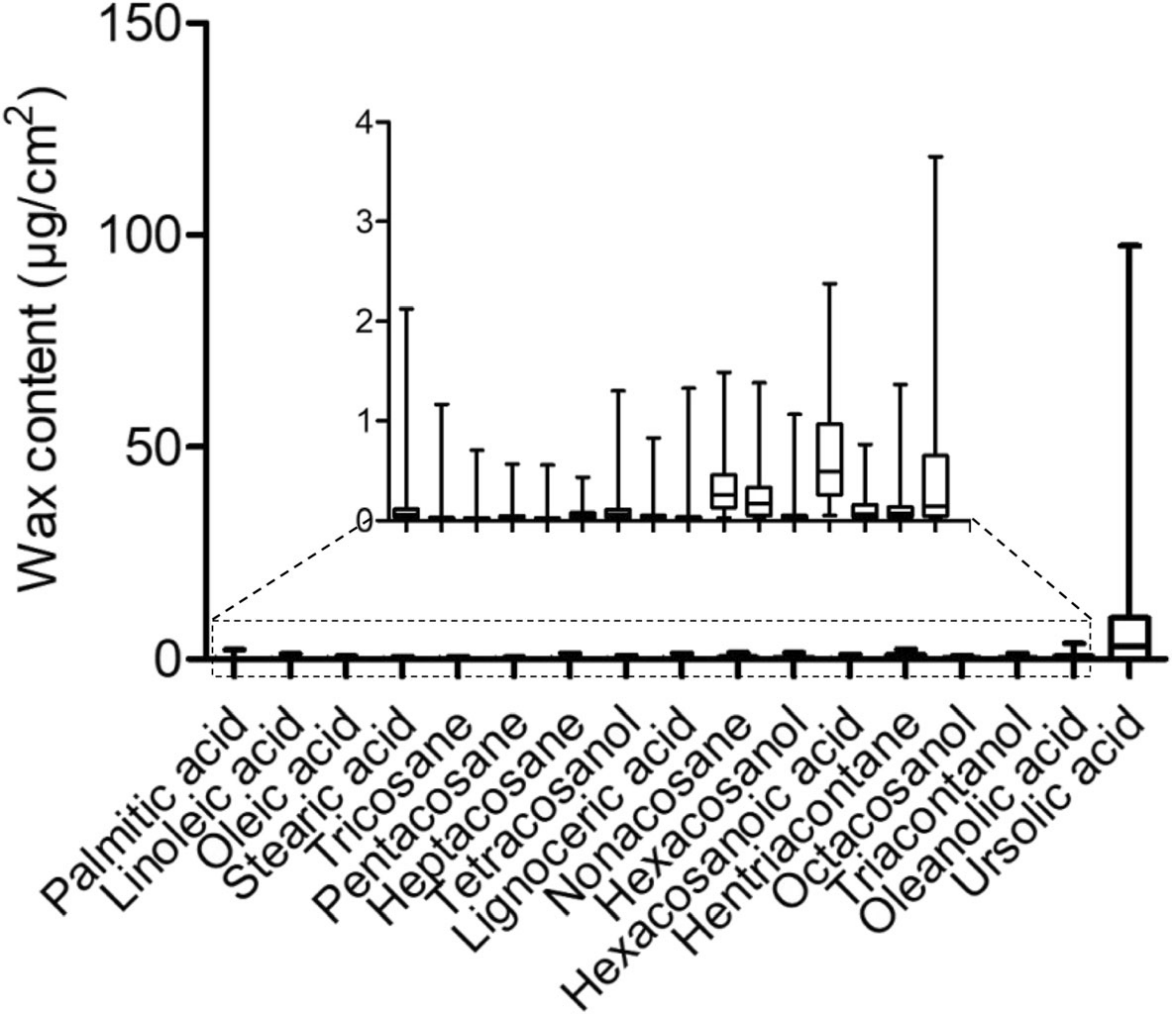
The distribution of the content of leaf wax components from 123 apple accessions. Bars are the content distribution of 123 apple accessions in each component

The wax contents of fresh leaves of 123 apple accessions were analyzed by gas chromatography-tandem mass spectrometry (GC-MS). These 123 apple accessions included 107 *Malus domestica* and 16 *M. sieversii*, of which four accessions were subspecies of M. sieversii f. neidzwetzkyana (Supplementary Table 1). Seventeen components in apple leaves were characterized, including alcohols, alkanes, fatty acids, and terpenes (Supplementary Table 2). The correlations among these 17 components were analyzed by Pearson correlation analysis (Fig. 1). The results showed that the first nine components were strongly correlated with each other. However, components 10, 11, 13, 16 and 17 were not strongly correlated with most other components (Fig. 1). In addition, the results showed that the contents of ursolic acid, oleanolic acid, hentriacontane and nonacosane were relatively high among the 17 components within the wax of apple leaves (Fig. 2).

### Population structure and linkage disequilibrium

We sequenced the genomes of the 123 *Malus* accessions using the Illumina HiSeq 4000 sequencing platform, and a total of 803 Gb of raw sequence data were obtained. Trimming of low-quality reads and removing adapter sequences resulted in an average coverage of clean sequence data of approximately 16-fold for each accession. These sequences were aligned to a reference genome and used for calling of single nucleotide polymorphisms (SNPs). A total of 5.94 million SNPs were obtained with a missing rate less than 0.1 and a minor allele frequency (MAF) of at least 0.05. These SNPs were used for subsequent analyses.

**Fig. 3:**
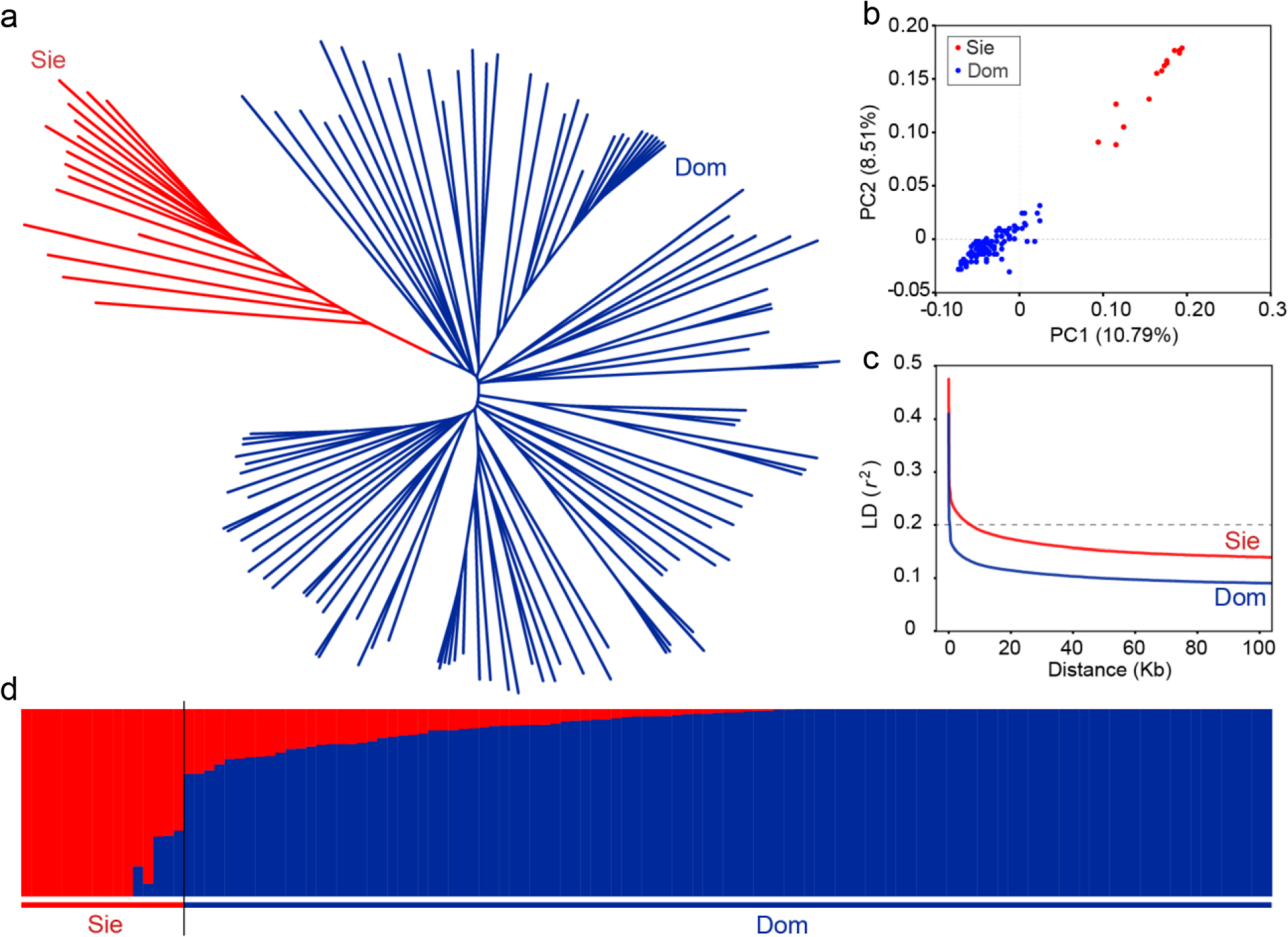
Population structure and linkage disequilibrium (LD) decay of apple genomes. Sie, *M. sieversii* accessions; Dom, *M. domestica* accessions. **a** Neighbor-joining phylogenetic tree of the 123 *Malus* accessions. **b** Principal component analysis (PCA) of the 123 apple accessions. **c** LD decay measured as the squared correlation coefficient (*r*^2^) by pairwise distance in Sie and Dom subgroups. **d** Admixture analysis of all apple accessions. The length of each colored segment represents the proportion of the individual genome inferred from ancestral populations (K = 2)

Excluding SNPs with high linkage disequilibrium (LD) (*r*^2^ > 0.5), a subset of 115,802 SNPs, which were evenly distributed over the entire *M. domestica* GDDH13 genome, was used for population structure estimation. We performed phylogenetic tree construction of these 123 *Malus* accessions. The results suggested that they were divided into two major subpopulations (Fig. 3a), which is consistent with the known evolutionary history of the domesticated apple (Cornille et al. 2014; Duan et al. 2017).

Principal component analysis (PCA) was applied to simplify and summarize the genetic marker variation of the 123 accessions. The first three components of PCA (Fig. 3b) suggested a degree of genetic divergence in the two subpopulations (*M. sieversii* and *M. domestica* accessions). To further understand the population structure, we estimated ancestry proportions using a model-based method for each accession. For K = 2, *M. domestica* and *M. sieversii* accessions were clearly separated from each other (Fig. 3c).

The LD decayed rapidly in the two subpopulations, and the decay was faster in domesticated apple than in its ancestral species, *M. sieversii*. This relative rapidity of decay of LD in domesticated apples implies that the domestication bottleneck in cultivated apple is weak. In addition, because of the rapidly decaying LD, a higher density of genetic markers is required for association analyses.

### GWAS analysis of 17 components of leaf wax

In order to explore the genetic basis of 17 components of wax in leaves, GWAS was performed by using the wax content data of the 123 apple accessions. According to the quantile-quantile (Q-Q) plot of the GWAS results, the *p* value observed in the lower left corner of the Q-Q plot was consistent with the expected *p* value. Therefore, we are confident that the *p* value obtained by the current statistical model conforms to the expected *p* value and that the statistical model is reasonable. In the upper right corner of the Q-Q plot are sites with high significance; these candidate sites are potentially associated with components (Supplementary Figs. 1 and 2).

The 17 components of the wax of apple leaves were analyzed to identify the key genes controlling the synthesis and transport of wax. Four components were of particular focus because they are of relatively high content within leaf wax. These four components are ursolic acid, oleanolic acid, hentriacontane and nonacosane. However, the GWAS results on ursolic acid were not further analyzed due to the large variance of the ursolic acid content. Therefore, our analysis focused on oleanolic acid, hentriacontane and nonacosane (Fig. 2).

The functions of genes associated with significant SNPs in the GWAS analysis were determined by Gene Ontology (GO) annotation, and GO annotation was similarly conducted for the three components. For these three components, the annotated genes were involved in biological processes, cellular components and molecular functions (Supplementary Figs. 3, 4, and 5). With regard to leaf wax nonacosane, 17 genes identified were associated with plant epidermis morphogenesis within the biological process category, and 12 identified genes were involved in the membrane protein complex in the cellular component category (Supplementary Fig. 3, Supplementary Table 3). For hentriacontane, some genes identified were associated with negative regulation of the developmental process and the fatty acid derivative biosynthetic process within the biological process category. Other genes were involved in transferase complexes and membrane protein complexes within cellular components (Supplementary Fig. 4, Supplementary Table 4). For oleanolic acid, some genes were involved in secondary metabolite biosynthetic processes, coenzyme biosynthetic processes, phenylpropanoid biosynthetic processes and phenylpropanoid metabolic processes in the biological process category (Supplementary Fig. 5, Supplementary Table 5).

### Candidate genes for wax biosynthesis

**Fig. 4:**
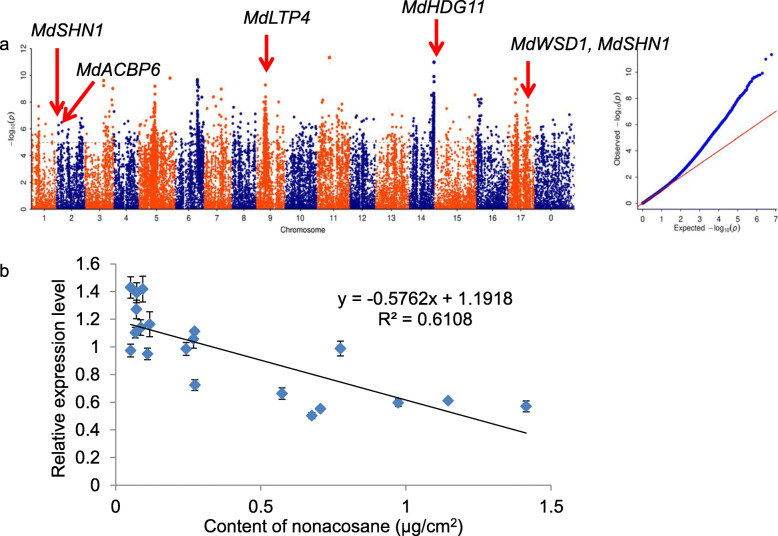
The GWAS result and GO annotation of nonacosane. **a** The GWAS result of nonacosane. A Manhattan plot of nonacosane is on the left. The chromosome number is plotted on the X-axis, and the -log_10_ value of the *p-*value is plotted on Y-axis. The dotted lines indicate -log_10_(*p*) = 5. A quantile-quantile (Q-Q) plot of nonacosane is on the right. The expected -log_10_ value of the *p-*value is on the X-axis and the observed -log_10_ value of the *p-*value is on the Y-axis. **b** Relationships of nonacosane content with expression of *MdACBP6* in apple leaves (*n* = 20). The data were analyzed by linear regression. Each point represents an accession, and these data include accessions with high and low nonacosane content, expressed as μg/cm^2^. Error bars indicate standard deviation (*n* = 3)

**Fig. 5:**
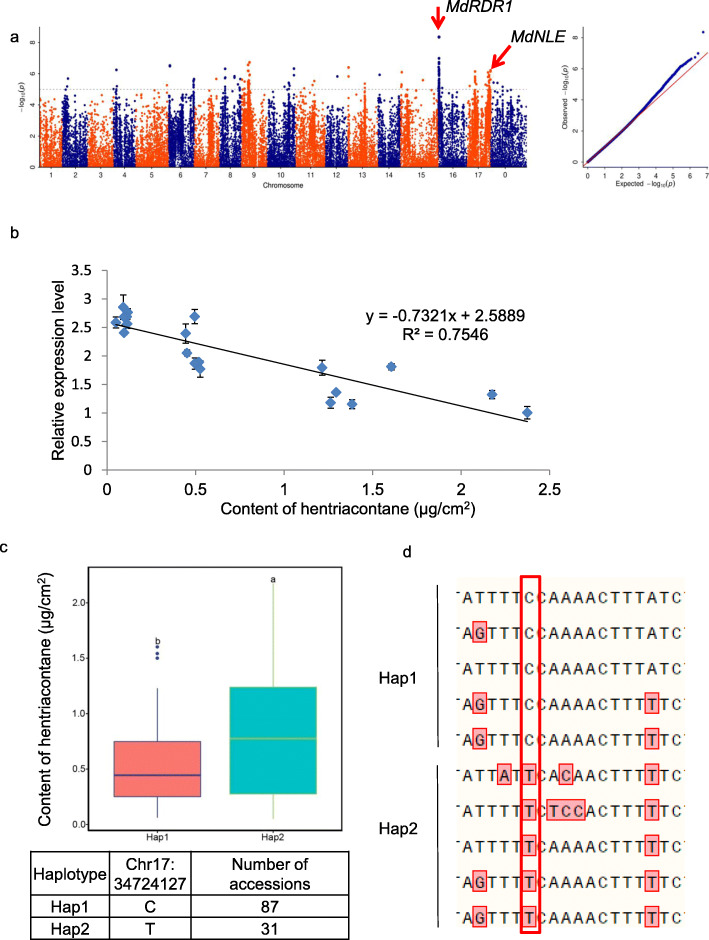
The GWAS result and GO annotation of hentriacontane. **a** The GWAS result of hentriacontane. A Manhattan plot of hentriacontane is on the left. The chromosome number is plotted on the X-axis, and the -log_10_ value of the *p-*value is plotted on Y-axis. The dotted lines indicate -log_10_(*p*) = 5. A quantile-quantile (Q-Q) plot of hentriacontane is on the right. The expected -log_10_ value of the *p-*value is on the X-axis and the observed -log_10_ value of the *p-*value is on the Y-axis. **b** Relationships of hentriacontane content with expression of *MdNLE* in apple leaves (*n* = 20). The data were analyzed by linear regression. Each point represents an accession, and these data include accessions with high and low hentriacontane content, expressed as μg/cm^2^. Error bars indicate standard deviation (*n* = 3). **c** Comparative analyses of *MdNLE* between the low and high hentriacontane haplotypes according to the most significant SNP of the *MdNLE* promoter. Boxplot shows the content of hentriacontane of each haplotype. The box expresses the upper, and the median and lower quartiles, and the dots represent extremes. Different letters indicate significant differences at *p* < 0.05 according to one-way analysis of variance (ANOVA). The table represents the location of the most significant SNP of *MdNLE* promoter and the accessions number corresponding to each haplotype. **d** SNP was verified by Sanger sequencing and each line is a mixture of four accessions. The red box is the location of the SNP

**Fig. 6:**
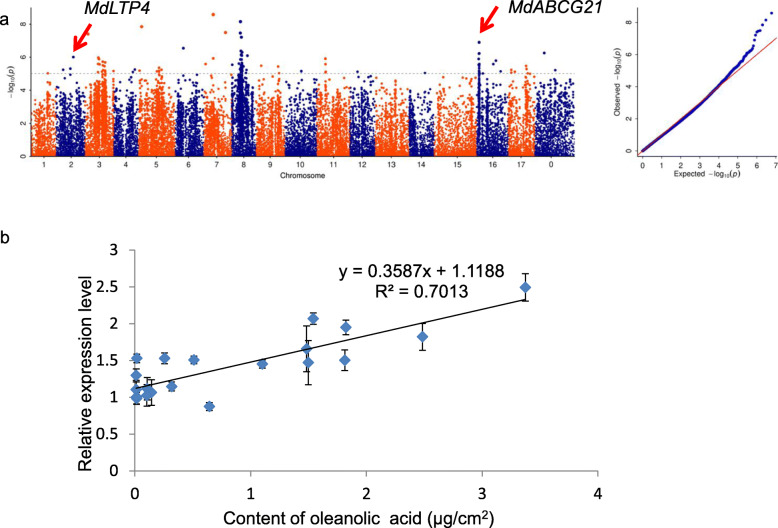
The GWAS result and GO annotation of oleanolic acid. **a** The GWAS result of oleanolic acid. A Manhattan plot of oleanolic acid is on the left. The chromosome number is plotted on the X-axis, and the -log_10_ value of the *p-*value is plotted on the Y-axis. The dotted lines indicate -log_10_(*p*) = 5. A quantile-quantile (Q-Q) plot of oleanolic acid is on the right. The expected -log_10_ value of the *p-*value is on the X-axis and the observed -log_10_ value of the *p-*value is on Y-axis. **b** Relationships of oleanolic acid content with expression of *MdABCG21* in apple leaves (*n* = 20). The data were analyzed by linear regression. Each point represents an accession, and these data include accessions with high and low oleanolic acid content, expressed as μg/cm^2^. Error bars indicate standard deviation (*n* = 3)

Among the 17 components, three components were emphasized. Some known and new genes involved in the biosynthesis of wax in apple leaves were found (Figs. 4a, 5a, and 6a).

In the GWAS of leaf nonacosane, several significant SNPs were associated with some of the reported genes, including *MdSHN1*, *MdLTP4* (*LIPID TRANSFER PROTEIN4*) and *MdWSD1* (Cameron et al. 2006; Li et al. 2008; Oshima et al. 2013). One significant SNP was associated with gene MD02G1030900 on chromosome 2. This gene is homologous to *AtSHN1*. The product of *AtSHN1*, a member of the ERF/AP2 transcription factor family, induces the production of wax by involving in the alkane synthesis pathway. Over-expression of *AtSHN1* makes leaves smooth and enhances drought tolerance of Arabidopsis (Aharoni et al. 2004). Twelve significant SNPs were located in MD09G1144900, which is homologous to *AtLTP4*. Three of these significant SNPs were located in the promoter region of the gene. The lipid transferase proteins encoded by *AtLTP4* localize to the endoplasmic reticulum and bind fatty acids and acyl CoA esters in the process of lipid transport (Cameron et al. 2006). Another significant SNP was located in the promoter of a known wax synthesis-related gene, MD17G1237100, which is a homolog of *AtWSD1-LIKE*. This gene belongs to a member of the bifunctional wax ester synthase/diacylglycerol acyltransferase gene family (Li et al. 2008). Additionally, a significant SNP related to nonacosane was located in MD02G1087600, which is a homolog of the Arabidopsis *ACYL-COA-BINDING PROTEIN6* (*AtACBP6*). To further verify the role of *MdACBP6* in the synthesis of nonacosane, expression levels of *MdACBP6* across 20 accessions were detected by real-time quantitative PCR (RT-qPCR). The results showed that the expression level of *MdACBP6* was negatively correlated with the content of nonacosane, suggesting that *MdACBP6* negatively regulates the accumulation of this component of leaf wax. There was a strong linear relationship between the content of nonacosane of leaf wax and the expression level of *MdACBP6* (*r*^2^ = 0.611) (Fig. 4a and b, Supplementary Table 8). However, haplotype analysis showed no significant difference in wax content between the two haplotypes (Supplementary Fig. 7), indicating that the difference in expression level of *MdACBP6* may be due to epigenetic modifications on promoters in different accessions.

Regarding hentriacontane, two significant SNPs located in the promoter of MD16G1017500 which is a homolog of the Arabidopsis gene that encodes *RNA-DEPENDENT RNA POLYMERASE1* (*AtRDR1*). Studies by Lam et al. (2012) have shown that the product of the *AtRDR1* gene, together with *SUPPRESSOR OF GENE SILENCING3* (*AtSGS3*), regulates the production of epidermal wax in Arabidopsis by regulating the expression level of *AtCER3*. Additionally, four prominent SNPs were located in MD17G1287700. One of these four SNPs was the most prominent SNP on chromosome 17. MD17G1287700 is a homolog of Arabidopsis *NOTCHLESS* (*AtNLE*), which encodes a WD-40 repeat family protein. To further verify the role of *MdNLE* in the synthesis of hentriacontane, expression levels of *MdNLE* across 20 accessions were detected by RT-qPCR. These results showed that the expression level of *MdNLE* was negatively correlated with the content of hentriacontane, suggesting that *MdNLE* may negatively regulate the accumulation of hentriacontane in leaf wax. There was a strong linear relationship between the content of hentriacontane of leaf wax and the expression level of *MdNLE* (*r*^2^ = 0.755) (Fig. 5a and b, Supplementary Table 9). In order to investigate whether natural variations in *MdNLE* promoter contribute to the variation of its expression level, we performed haplotype analysis on *MdNLE* promoter. Results showed that the two haplotypes were separated based on the most prominent SNP on *MdNLE* promoter, with the Hap1 and Hap2 groups containing 87 and 31 accessions, respectively (Fig. 5c). Varieties carrying Hap1 showed a lower hentriacontane content than Hap2 varieties. We verified the existence of this SNP by Sanger sequencing with 40 accessions (Fig. 5d). The result indicated that the natural variation of *MdNLE* promoter may be the main effect SNP that causes the difference of hentriacontane content in different accessions.

With respect to the oleanolic acid, one SNP, which is the most significant SNP on chromosome 2, located in MD02G1211600 which is a homolog of *AtLTP4*. Moreover, on chromosome 16, one prominent SNP located in MD16G1050400, a homolog of an Arabidopsis gene that encodes an *ATP-BINDING CASSETTE G21* (*AtABCG21*) transporter. Interestingly, this SNP, which is located in the coding sequence of *MdABCG21*, is the most prominent SNP on chromosome 16. To further analyze the potential role of *MdABCG21* in the synthesis of oleanolic acid, expression levels of *MdABCG21* across 20 accessions were detected by RT-qPCR. There was a strong positive linear relationship between the content of oleanolic acid and the expression level of *MdABCG21* (*r*^2^ = 0.701), suggesting that *MdABCG21* may be positively involved in the accumulation of oleanolic acid in leaf wax (Fig. 6a and b**,** Supplementary Table 10). Nevertheless, haplotype analysis showed no significant difference in wax content between the two haplotypes (Supplementary Fig. 8), indicating epigenetic modifications on promoters might be involved.

## Discussion

In this study, 17 leaf wax components of 123 apple accessions were quantified (Supplementary Table 2). By exploring the correlations among these 17 wax components, we found that the first 9 components were strongly correlated, with correlation coefficients of 0.6 to 0.9, but the correlations of components 10, 13, 16, and 17 with other components were relatively weak (Fig. 1). Because the longest chain within the first nine components has 27 carbons, the strong correlation here may due to the fact that the FAE complex can only extend the carbon chain to 28 carbons (Haslam et al. 2015; Haslam et al. 2012; Pascal et al. 2013). The weak correlations of components 10, 13 with most other components may be due to the fact that the pathways leading to the synthesis of these components are alkane synthesis pathways, and carbon chains of components in this pathway have more than 28 carbons. The weak correlations of components 16 and 17 with most other components may be due to the fact that Oleanolic acid and Ursolic acid are terpenoids (Kunst and Samuels 2003).

Using phenotypic data from the leaves of 123 apple accessions, GWAS was performed on the wax content to identify genes involved in synthesis and transport of wax. We focused on the 17 prominent wax components identified in the GC-MS analysis (Supplementary Figs. 1 and 2). GO analysis was performed for all genes above threshold lines in GWAS of 17. These annotated genes were found to be involved in biological processes, cellular components, and molecular functions among the GO categories. The analysis indicated that 113 of the identified genes were associated with leaf development within biological processes, 79 genes were involved in vesicles within cellular components, 60 genes were associated with the Golgi subcompartment of cellular components and 52 genes were involved in the plasma membrane aspect of cellular components (Supplementary Fig. 6, Supplementary Table 6). The results are consistent with some hypotheses that wax transports from the ER to the plasma membrane as it is a hydrophobic substance and requires transport through the aqueous cytoplasm.

Three components were highlighted among the 17 wax components, including nonacosane, hentriacontane, and oleanolic acid. Previous studies have shown that the increase of leaf wax content under drought conditions is mainly due to the increase of long-chain alkanes, including nonacosane and hentriacontane. It shows that these two components of leaf wax play an important role in drought stress tolerance (Kosma et al. 2009; Panikashvili et al. 2007). Because of the key role played by the alkanes, it is of great significance to study these two components in apple leaf wax. In the GWAS of these three components, some genes associated with significant SNPs are known to be involved in wax synthesis and transport, including *MdSHN1*, *MdLTP4*, *MdWSD1*, and *MdRDR1* (Cameron et al. 2006; Li et al. 2008; Lam et al. 2012; Oshima et al. 2013). We also identified some novel genes associated with significant SNPs, including *MdACBP6*, *MdNLE,* and *MdABCG21* **(**Figs. 4, 5 and 6**)**. The SNPs associated with these genes are located in the promoter, the coding sequence, downstream of the gene, or between genes. Notably, 20 significant SNPs related to nonacosane were found in MD14G1243300 on chromosome 14. This gene is homologous to *HOMEODOMAIN GLABROUS11* (*HDG11)* in Arabidopsis, which belongs to the HD-ZIP IV family of homeobox-leucine zipper proteins. Previous studies have shown that the product of the *HDG11* gene regulates the development of leaf trichomes in Arabidopsis (Khosla et al. 2014), while leaf trichomes and epidermal waxes have same precursors (Hegebarth et al. 2017). These connections suggest that MdHDG11 may regulate the epidermal wax in apple leaves, but this needs further analysis. There are two nonsynonymous mutations among the 20 significant SNPs associated with MdHDG11; these two significant SNPs may provide the basis for molecular breeding (Fig. 4a, Supplementary Table 8). We also found significant differences in wax content between the two haplotypes based on the most significant SNP in the promoters of *MdLTP4*, *MdWSD1* and *MdRDR1* (Supplementary Fig. 9), implying the potential usage of these SNPs in breeding in the future. More importantly, we identified a significant SNP in *MdNLE* promoter (Fig. 5c). Haplotype analysis and Sanger sequencing with 40 samples verified this SNP, indicating that *MdNLE* may be a key locus affecting wax of apple leaves (Fig. 5d).

However, there was no significant difference in wax content between the two haplotypes of *MdACBP6* and *MdABCG21*, respectively (Supplementary Figs. 7 and 8). Studies have shown that dynamic chromatin environment has a great influence on gene expression in eukaryotic cells. Epigenetic modification, including chromatin accessibility and covalent modification of histone tails and DNA, can alter chromatin status to influence gene expression (He et al. 2015; Chang et al. 2020; Chen et al. 2020). Therefore, the difference in expression level of *MdACBP6* and *MdABCG21* may be due the epigenetic modification which affects the expression of these two genes needs to be further studied.

Studies have shown that wax synthesis is mainly controlled by regulation at transcriptional and post-translational levels. Therefore, various genes associated with significant SNPs in other components should also be further studied. These SNPs may perform different functions depending on their location on the associated genes. Taken together, our results provide a new direction for molecular breeding of apple trees with altered wax content.

## Conclusion

In conclusion, we performed GWAS on leaf waxes of 123 *Malus* accessions. Seventeen major wax components and their contents were detected by GC-MS, and 5.9 million high-quality SNPs were identified by using whole-genome sequencing. Some important candidate genes, including MdSHN1, MdLTP4, MdWSD1, MdACBP6, MdRDR1, MdNLE, and MdABCG21, were identified which may play critical roles in the synthesis and transport of leaf waxes. These results provide hints for the mechanisms of wax synthesis and transport in apple leaves and for future resistance breeding.

## Materials and methods

### Samples for testing wax contents

Leaves of apple accessions (*n* = 123) were collected from the Horticulture Experimental Station of Northwest A&F University, Yangling, Shaanxi Province, China. Six leaves were collected from 3 trees of each accession.

### Cuticular wax analysis of apple leaves

Wax extraction and measurement were described by Wang et al. (2018) with modifications. Briefly, leaves were steeped in 20 mL chloroform for 60 s, with 20 μl n-tetracosane (1 μg/μl) added as an internal standard. The chloroform extract containing wax was filtered through a 0.45 μm organic filter and dried with a stream of nitrogen. The residue was mixed with 40 μl pyridine and 40 μl *N, O*-bis (trimethylsilyl) trifluoroacetamide (BSTF) in a water bath at 70 °C for 60 min and then dried with a stream of nitrogen. The derivatized residue was redissolved in 1 mL chloroform and filtered through a 0.22 μm organic membrane. The extract was analyzed with GC-MS on a DB-5 MS column coupled with a Trace GC ULTRA/ISQ MS detector (Thermo Scientific, USA). Helium was used as the carrier gas of the column and split injection (10:1) was performed at 250 °C. The oven temperature was increased to 50 °C and kept at 50 °C for 2 min, increased at 20 °C min^− 1^ to 240 °C and kept at 240 °C for 2 min, and increased at 1.5 °C min^− 1^ to 320 °C and kept at 320 °C for 15 min. The total wax content was expressed per unit area of the blade surface. Leaf area was measured with an LI-3000C Portable Area Meter (LI-COR Biosciences).

The wax components were characterized by mass spectrometry combined with the mixed standard including C7-C40 saturated alkanes (CRM, Sigma, 49,452-U). The conversion of GC-MS amount data to the wax content was performed by the following formula:

$$ \mathrm{Sample}\ \mathrm{wax}\ \mathrm{component}\ \mathrm{content}=\left(\mathrm{A}\ast \mathrm{B}\right)/\left(\mathrm{C}\ast \mathrm{D}\right). $$

A: Internal standard content (μg).

B: Peak area of sample.

C: Peak area of internal standard.

D: Leaf area (cm^2^).

### Read alignment and SNP calling

The sequenced reads were derived from the apple genus resequecing dataset (Chen et al. 2021). The 123 *Malus* accessions which were used to analyze cuticular wax content were sequenced by whole-genome high-depth sequencing technology. Raw reads were trimmed using Trimmomatic (version 0.38) (Bolger, Lohse, & Usadel, 2014) with the following parameters: LEADING:3 TRAILING:3 SLIDINGWINDOW:4:15 MINLEN:40 TOPHRED33. Cleaned reads were mapped to the GDDH13 reference genome using BWA-MEM (version 0.7.17) (Li and Durbin 2009) with default parameters. Duplicated reads were marked and removed with Picard tools (version 2.1.1) (http://broadinstitute.github.io/picard).

SNP detection was performed using GATK (version 3.8) (McKenna et al. 2010) with “Best Practices” workflow for variant calling. SNPs that did not meet the following criteria were excluded: (a) biallelic alleles; (b) a total read depth greater than 150 and less than 3000; (c) minor allele frequency of at least 0.05; and (d) a maximum missing rate less than or equal to 0.1.

### Population structure and LD analysis

For a more reasonable inference of the population structure, a total of 115,802 SNPs were randomly selected for population structure analysis. To understand the phylogenetic relationship, a distance matrix was generated by Plink (Chang et al. 2015) with parameter of ‘--distance-matrix’ and then a phylogeny tree was constructed using MEGA (version 10.2.4) (Kumar et al. 2018) with ‘Neighbor-Joining’ statistical method. PCA was performed using GCTA (version 1.01) (Yang et al. 2011). Population structure was investigated using ADMIXTURE (version 1.3) (Alexander and Lange 2011) which is a model-based clustering method for inferring population structure. We predefined genetic cluster (K) values from 2 to 6 and ran 20 replicates for each K to estimate the standard errors.

LD decay was calculated with PopLDdecay (version 3.41) (Zhang et al. 2019) with SNPs with a MAF of at least 0.05 and a maximum distance of 300 kb.

### GWAS analysis

An Efficient Mixed-Model Association program, EMMAX, was used for the association analysis (Kang et al. 2010). The first three PCA values (eigenvectors), which were derived from whole-genome SNPs, were used as fixed effects. The random effect was estimated by kinship among all accessions. An IBS kinship matrix was derived from all SNPs with the EMMAX-Kin program. The cutoff was *p* = 1 × 10^− 5^.

### Haplotype analysis

The significant SNPs in 2-kb promoter region of candidate genes were analyzed to haplotype phasing. Haplotypes in the LD blocks were calculated using the Haploview software with main parameters “-blockoutput GAB -blockCutHighCI 0.95 -blockCutLowCI 0.05 -minMAF 0.05”. Then, the accessions with wax content were divided into groups according to haplotypes, and Statistical analysis was performed using one-way analysis of variance (ANOVA) followed by Duncan’s multiple range test.

### Total RNA extraction and RT-qPCR analysis

Total RNA was extracted from apple leaves by using CTAB and DNase I treatment. A sample of 1 μg of total RNA was reverse transcribed into cDNA using HiScriptIIQ RT SuperMix for qPCR (+gDNA wiper) (Vazyme Biotech Co, Nanjing, China). RT-qPCR was performed using ChamQ SYBR qPCR Master Mix (Vazyme Biotech Co, Nanjing, China) with a Bio-Rad CFX real-time PCR detector. *MdMDH* was used as an internal reference gene for RT-qPCR.

### GO (Gene Ontology) analysis

The SNPs associated with GWAS were annotated according to the apple reference genome GDDH13 (https://iris.angers.inra.fr/gddh13/) by using ANNOVAR software to obtain mutation sites and the identities of adjacent genes approximately 50 kb upstream and downstream of each SNP (Wang et al. 2010). BLASTP in NCBI-BLAST 2.9.0+ was used with the following key parameters. The E-value was equal to 1 × 10^− 5^, the suspension value of the best hit algorithm was 0.25, the edge value of the score of the best hit algorithm was 0.05, and protein sequences from The Arabidopsis Information Resource version 10 (TAIR10) were used as queries in BLAST searches. Genes associated with significant SNP were analyzed for GO enrichment by using the R package clusterProfiler (Yu et al. 2012).

## Supplementary Information

**Additional file 1: Supplementary Table 1.** Details of all apple accessions**. Supplementary Table 2.** The contents of 17 wax components in leaves of 123 apple accessions (Each data is average of three biological replicates, unit: μg/cm2). **Supplementary Table 3.** Details of GO annotation of genes above the threshold line associated with nonacosane. **Supplementary Table 4.** Details of GO annotation of genes above the threshold line associated with hentriacontane. **Supplementary Table 5.** Details of GO annotation of genes above the threshold line associated with oleanolic acid. **Supplementary Table 6.** Details of GO annotation of genes above the threshold line associated with all components. **Supplementary Table 7.** List of all qPCR primers. **Supplementary Table 8.** List of significant loci from GWAS of nonacosane. **Supplementary Table 9.** List of significant loci from GWAS of hentriacontane. **Supplementary Table 10.** List of significant loci from GWAS of oleanolic acid. **Supplementary Table 11.** Gene ID information of the candidate genes for nonacosane. **Supplementary Table 12.** Gene ID information of the candidate genes for hentriacontane. **Supplementary Table 13.** Gene ID information of the candidate genes for oleanolic acid. **Supplementary Table 14.** Sanger sequencing primers of *MdNLE* promoter.

**Additional file 2 **: **Supplementary Fig. 1.** The GWAS results of different wax components of apple leaves (Components 1 to 8). The GWAS results of different wax components of apple leaves. Manhattan plots of GWAS is on the left. The chromosome number is plotted on the X-axis and the -log_10_ value of the *p-*value is plotted on Y-axis. The dotted lines indicate -log_10_(*p*) = 5. Quantile-quantile (Q-Q) plots of GWAS is on the right. The expected -log_10_ value of the *p-*value is on the X-axis and the observed -log_10_ value of the *p-*value is on Y-axis. **Supplementary Fig. 2.** The GWAS results of different wax components of apple leaves (Components 9 to 17). The GWAS results of different wax components of apple leaves. Manhattan plots of GWAS is on the left. The chromosome number is plotted on the X-axis, and the -log_10_ value of the *p-*value is plotted on Y-axis. The dotted lines indicate -log_10_(*p*) = 5. Quantile-quantile (Q-Q) plots of GWAS is on the right. The expected -log_10_ value of the *p*-value is on the X-axis and the observed -log_10_ value of the p-value is on Y-axis. **Supplementary Fig. 3.** Gene Ontology (GO) annotation of all associated genes above the threshold line from GWAS result of nonacosane. **Supplementary Fig. 4.** Gene Ontology (GO) annotation of all associated genes above the threshold line from GWAS result of hentriacontane. **Supplementary Fig. 5.** Gene Ontology (GO) annotation of all associated genes above the threshold line from GWAS result of oleanolic acid. **Supplementary Fig. 6.** GO (Gene Ontology) annotation of all associated genes above the threshold line from GWAS results of all components. **Supplementary Fig. 7.** Comparative analyses of MdACBP6 between the low and high nonacosane haplotypes according to the most significant SNP of the MdACBP6 promoter. Boxplot shows the content of nonacosane of each haplotype. The box expresses the upper, and the median and lower quartiles, and the dots represent extremes. Different letters indicate significant differences at p < 0.05 according to one-way analysis of variance (ANOVA). The table represents the location of the most significant SNP of MdACBP6 promoter and the accessions number corresponding to each haplotype. **Supplementary Fig. 8.** Comparative analyses of MdABCG21 between the low and high oleanolic acid haplotypes according to the most significant SNP of the MdABCG21 promoter. Boxplot shows the content of oleanolic acid of each haplotype. The box expresses the upper, and the median and lower quartiles, and the dots represent extremes. Different letters indicate significant differences at p < 0.05 according to one-way analysis of variance (ANOVA). The table represents the location of the most significant SNP of MdABCG21 promoter and the accessions number corresponding to each haplotype. **Supplementary Fig. 9.** Comparative analyses of MdLTP4, MdWSD1, and MdRDR1 between the low and high wax haplotypes according to the most significant SNP of their promoters. Boxplot shows the content of wax of each haplotype. The box expresses the upper, the median, and lower quartiles, and the dots represent extremes. Different letters indicate significant differences at p < 0.05 according to one-way analysis of variance (ANOVA). The table represents the location of the most significant SNP of their promoters and the accessions number corresponding to each haplotype.

## Data Availability

Whole genome sequences data of 123 *Malus* accessions was submitted to the Sequence Read Archive (SRA) database under the accession numbers PRJNA354212.

## Abbreviations

GC-MS: Gas chromatography-tandem mass spectrometry
SNPs: Single nucleotide polymorphisms
GWAS: Genome-wide association study
VLCFAs: Very long chain fatty acids
malonyl-ACP: Malonyl-acyl carrier protein
FAS: Fatty acid synthase
LACS: Long-chain acyl coenzyme A synthetase
ACC: Acetyl-CoA carboxylase
FAE: Fatty acid elongase
KCS: *β*-Ketoacyl-CoA synthase
KCR: *β*-Ketoacyl-CoA reductase
HCD: *β*-Hydroxyacyl-CoA dehydratase
ECR: Enoyl-CoA reductase
ACBPs: Acyl-CoA binding proteins
ACB: Acyl CoA binding
LTPs: Lipid transfer proteins
AP2: APETALA2
ERF/EREBP: Ethylene-responsive element binding factor
MAF: Minor allele frequency
LD: Linkage disequilibrium
PCA: Principal component analysis
Q-Q plot: Quantile-quantile plot
GO: Gene Ontology
RT-qPCR: Real-time quantitative PCR
