# Supplementary material for: Genome-wide association study (GWAS) of leaf wax components of apple
Source: Stress Biol. 2021 Nov 18;1(1):13. doi: 10.1007/s44154-021-00012-3 (PMC10441854; doi:10.1007/s44154-021-00012-3)
Supplement: Supplementary file 2 — Additional file 2 : Supplementary Fig. 1. The GWAS results of different wax components of apple leaves (Components 1 to 8). The GWAS results of different wax components of apple leaves. Manhattan plots of GWAS is on the left. The chromosome number is plotted on the X-axis and the -log10 value of the p-value is plotted on Y-axis. The dotted lines indicate -log10(p) = 5. Quantile-quantile (Q-Q) plots of GWAS is on the right. The expected -log10 value of the p-value is on the X-axis and the observed -log10 value of the p-value is on Y-axis. Supplementary Fig. 2. The GWAS results of different wax components of apple leaves (Components 9 to 17). The GWAS results of different wax components of apple leaves. Manhattan plots of GWAS is on the left. The chromosome number is plotted on the X-axis, and the -log10 value of the p-value is plotted on Y-axis. The dotted lines indicate -log10(p) = 5. Quantile-quantile (Q-Q) plots of GWAS is on the right. The expected -log10 value of the p-value is on the X-axis and the observed -log10 value of the p-value is on Y-axis. Supplementary Fig. 3. Gene Ontology (GO) annotation of all associated genes above the threshold line from GWAS result of nonacosane. Supplementary Fig. 4. Gene Ontology (GO) annotation of all associated genes above the threshold line from GWAS result of hentriacontane. Supplementary Fig. 5. Gene Ontology (GO) annotation of all associated genes above the threshold line from GWAS result of oleanolic acid. Supplementary Fig. 6. GO (Gene Ontology) annotation of all associated genes above the threshold line from GWAS results of all components. Supplementary Fig. 7. Comparative analyses of MdACBP6 between the low and high nonacosane haplotypes according to the most significant SNP of the MdACBP6 promoter. Boxplot shows the content of nonacosane of each haplotype. The box expresses the upper, and the median and lower quartiles, and the dots represent extremes. Different letters indicate significant differences at p < 0.05 a [file 44154_2021_12_MOESM2_ESM.pptx]

## Slide 1
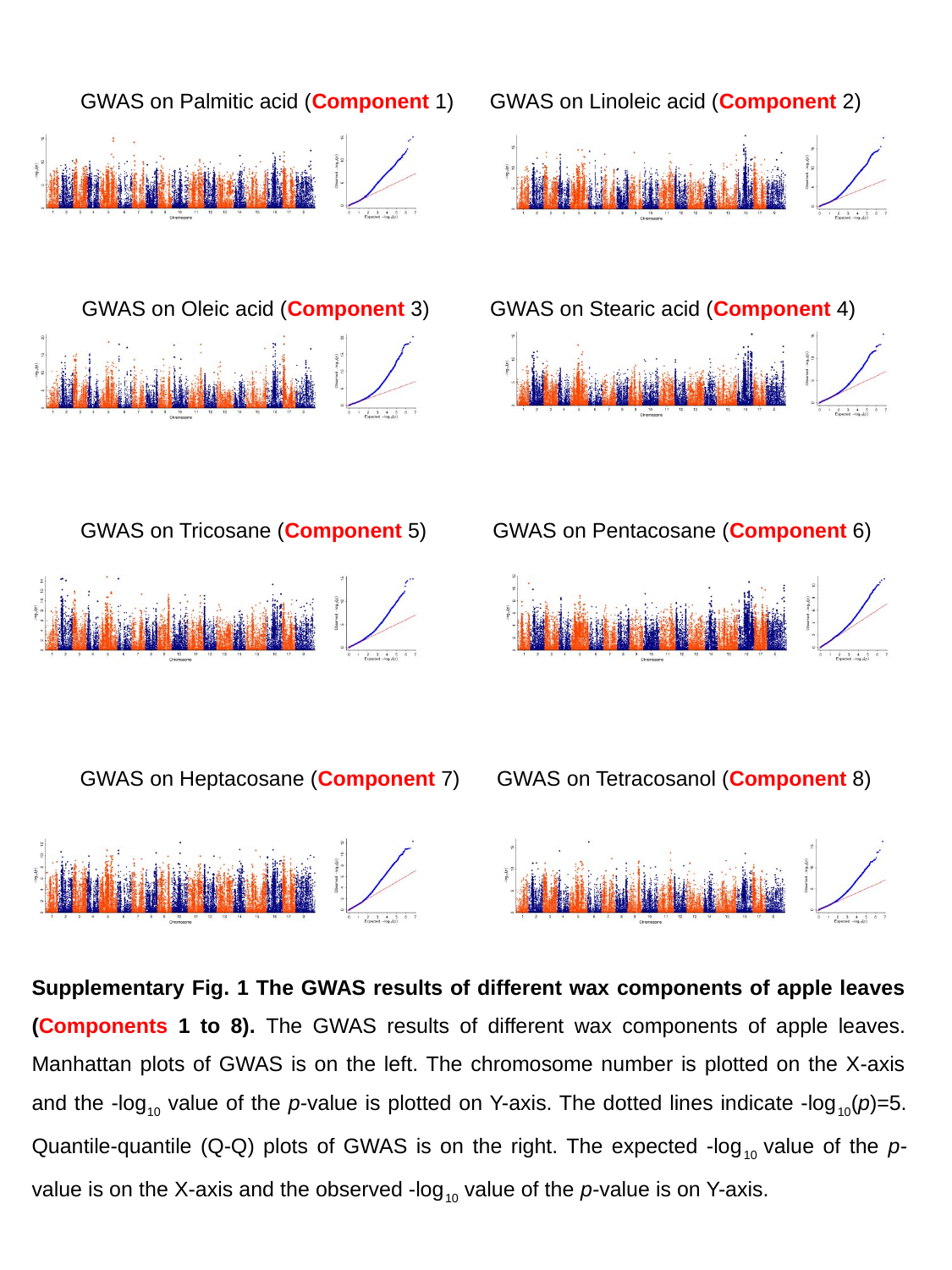

GWAS on Linoleic acid (Component 2)
GWAS on Palmitic acid (Component 1)
GWAS on Stearic acid (Component 4)
GWAS on Oleic acid (Component 3)
GWAS on Tricosane (Component 5)
GWAS on Pentacosane (Component 6)
GWAS on Tetracosanol (Component 8)
GWAS on Heptacosane (Component 7)
Supplementary Fig. 1 The GWAS results of different wax components of apple leaves (Components 1 to 8). The GWAS results of different wax components of apple leaves. Manhattan plots of GWAS is on the left. The chromosome number is plotted on the X-axis and the -log10 value of the p-value is plotted on Y-axis. The dotted lines indicate -log10(p)=5. Quantile-quantile (Q-Q) plots of GWAS is on the right. The expected -log10 value of the p-value is on the X-axis and the observed -log10 value of the p-value is on Y-axis.

## Slide 2
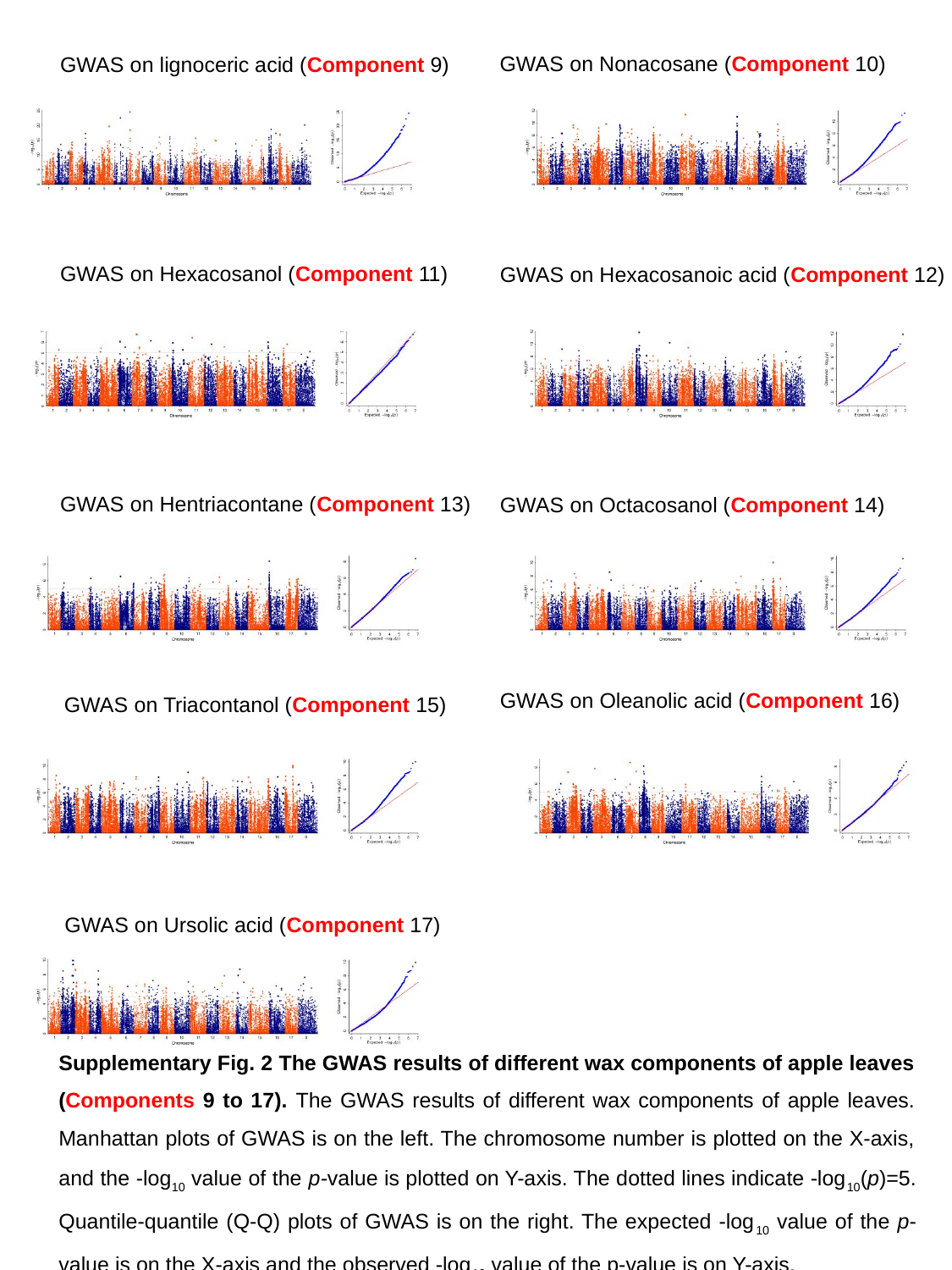

GWAS on Nonacosane (Component 10)
GWAS on lignoceric acid (Component 9)
GWAS on Hexacosanol (Component 11)
GWAS on Hexacosanoic acid (Component 12)
GWAS on Hentriacontane (Component 13)
GWAS on Octacosanol (Component 14)
GWAS on Oleanolic acid (Component 16)
GWAS on Triacontanol (Component 15)
GWAS on Ursolic acid (Component 17)
Supplementary Fig. 2 The GWAS results of different wax components of apple leaves (Components 9 to 17). The GWAS results of different wax components of apple leaves. Manhattan plots of GWAS is on the left. The chromosome number is plotted on the X-axis, and the -log10 value of the p-value is plotted on Y-axis. The dotted lines indicate -log10(p)=5. Quantile-quantile (Q-Q) plots of GWAS is on the right. The expected -log10 value of the p-value is on the X-axis and the observed -log10 value of the p-value is on Y-axis.

## Slide 3
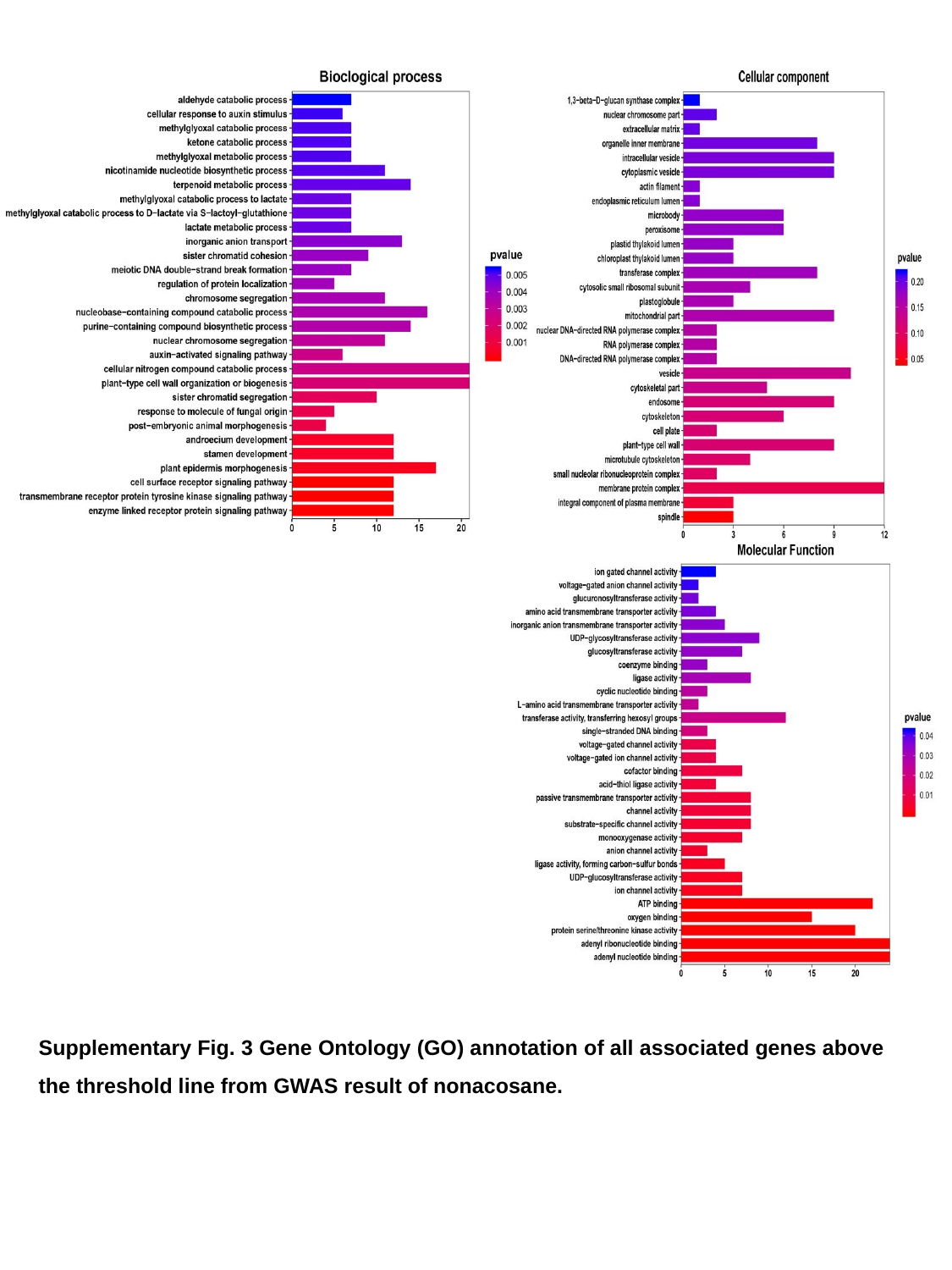

Supplementary Fig. 3 Gene Ontology (GO) annotation of all associated genes above the threshold line from GWAS result of nonacosane.

## Slide 4
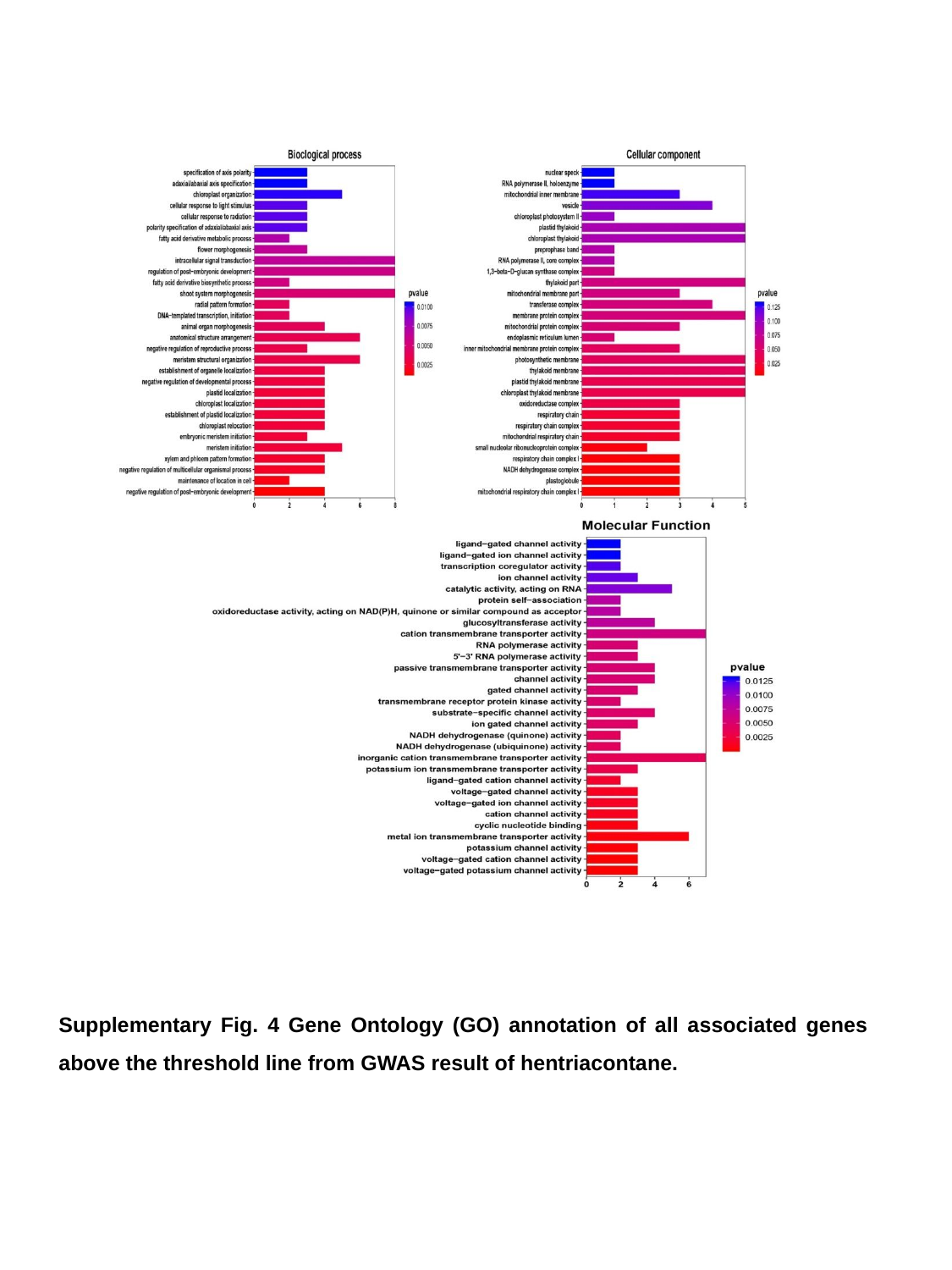

Supplementary Fig. 4 Gene Ontology (GO) annotation of all associated genes above the threshold line from GWAS result of hentriacontane.

## Slide 5
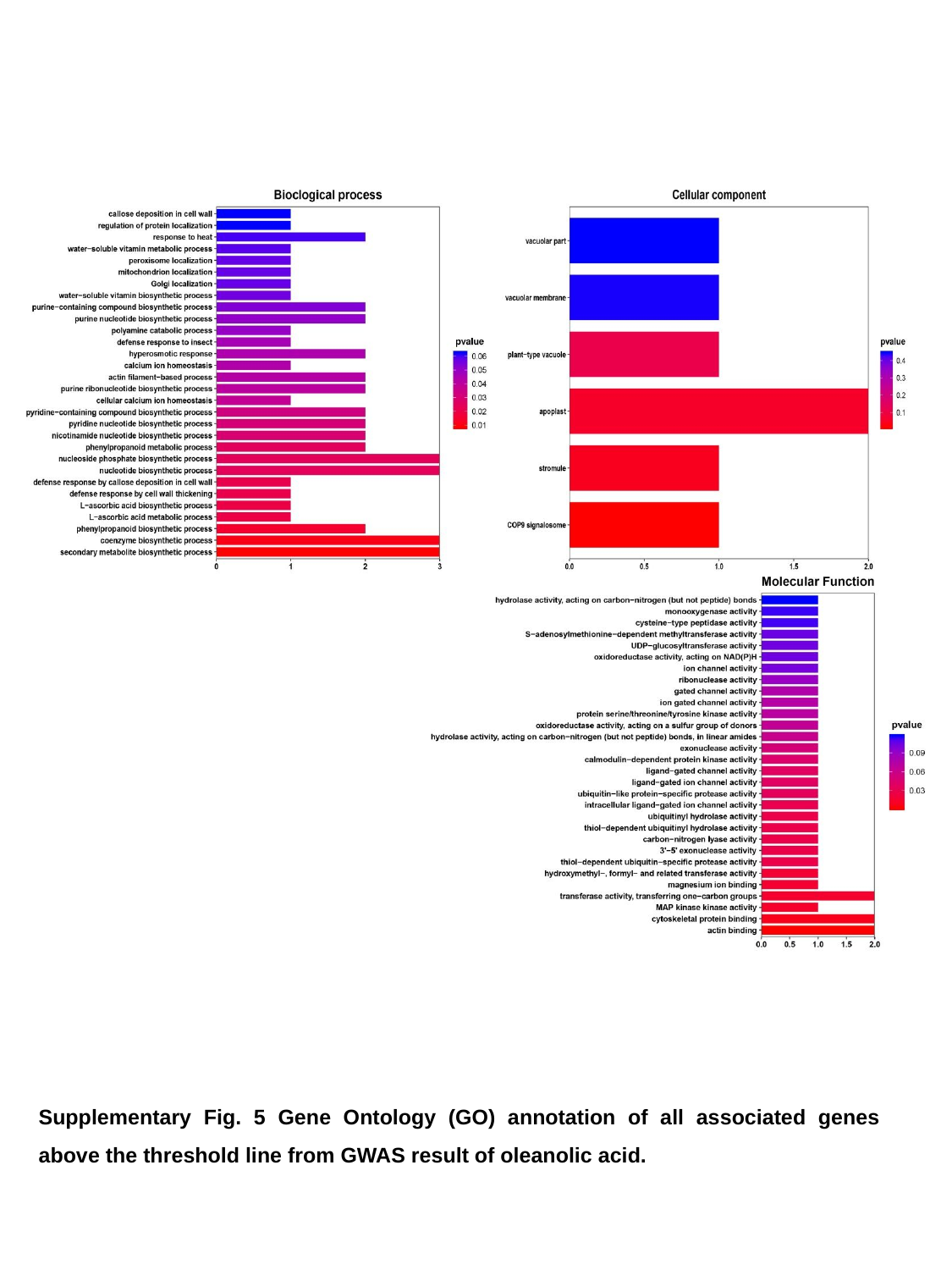

Supplementary Fig. 5 Gene Ontology (GO) annotation of all associated genes above the threshold line from GWAS result of oleanolic acid.

## Slide 6
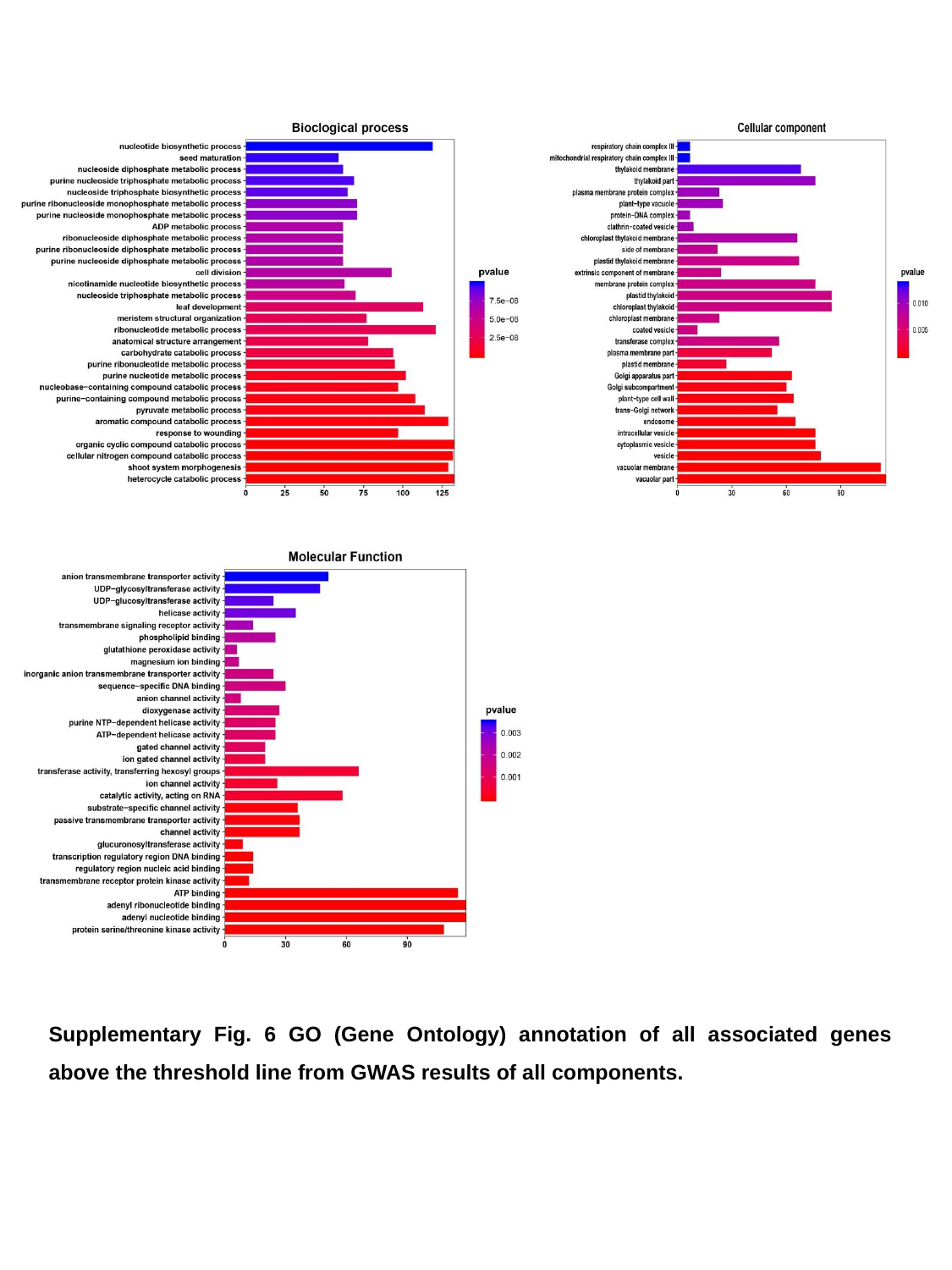

Supplementary Fig. 6 GO (Gene Ontology) annotation of all associated genes above the threshold line from GWAS results of all components.

## Slide 7
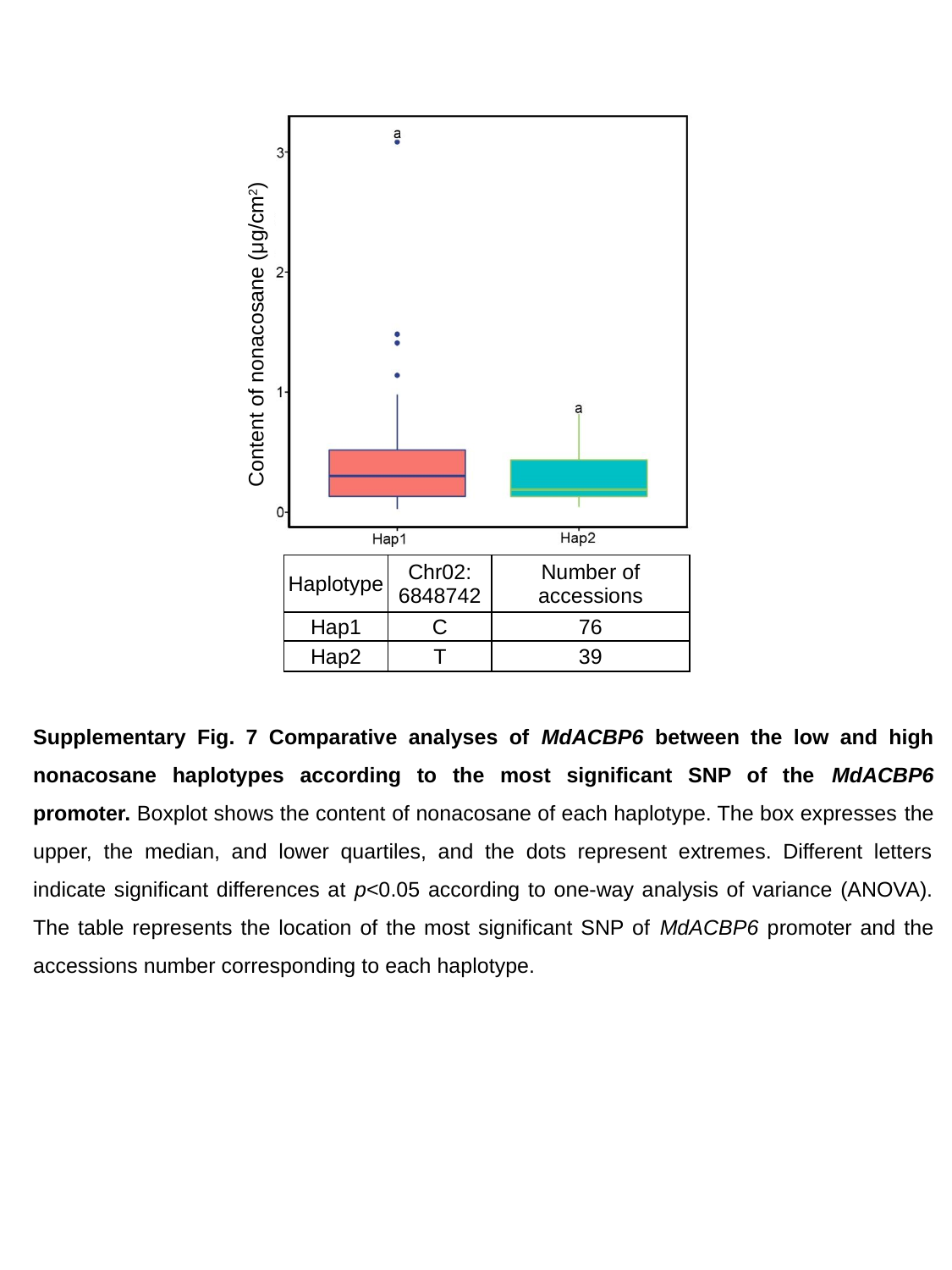

Content of nonacosane (μg/cm2)
| Haplotype | Chr02: 6848742 | Number of accessions |
| --- | --- | --- |
| Hap1 | C | 76 |
| Hap2 | T | 39 |
Supplementary Fig. 7 Comparative analyses of MdACBP6 between the low and high nonacosane haplotypes according to the most significant SNP of the MdACBP6 promoter. Boxplot shows the content of nonacosane of each haplotype. The box expresses the upper, the median, and lower quartiles, and the dots represent extremes. Different letters indicate significant differences at p<0.05 according to one-way analysis of variance (ANOVA). The table represents the location of the most significant SNP of MdACBP6 promoter and the accessions number corresponding to each haplotype.

## Slide 8
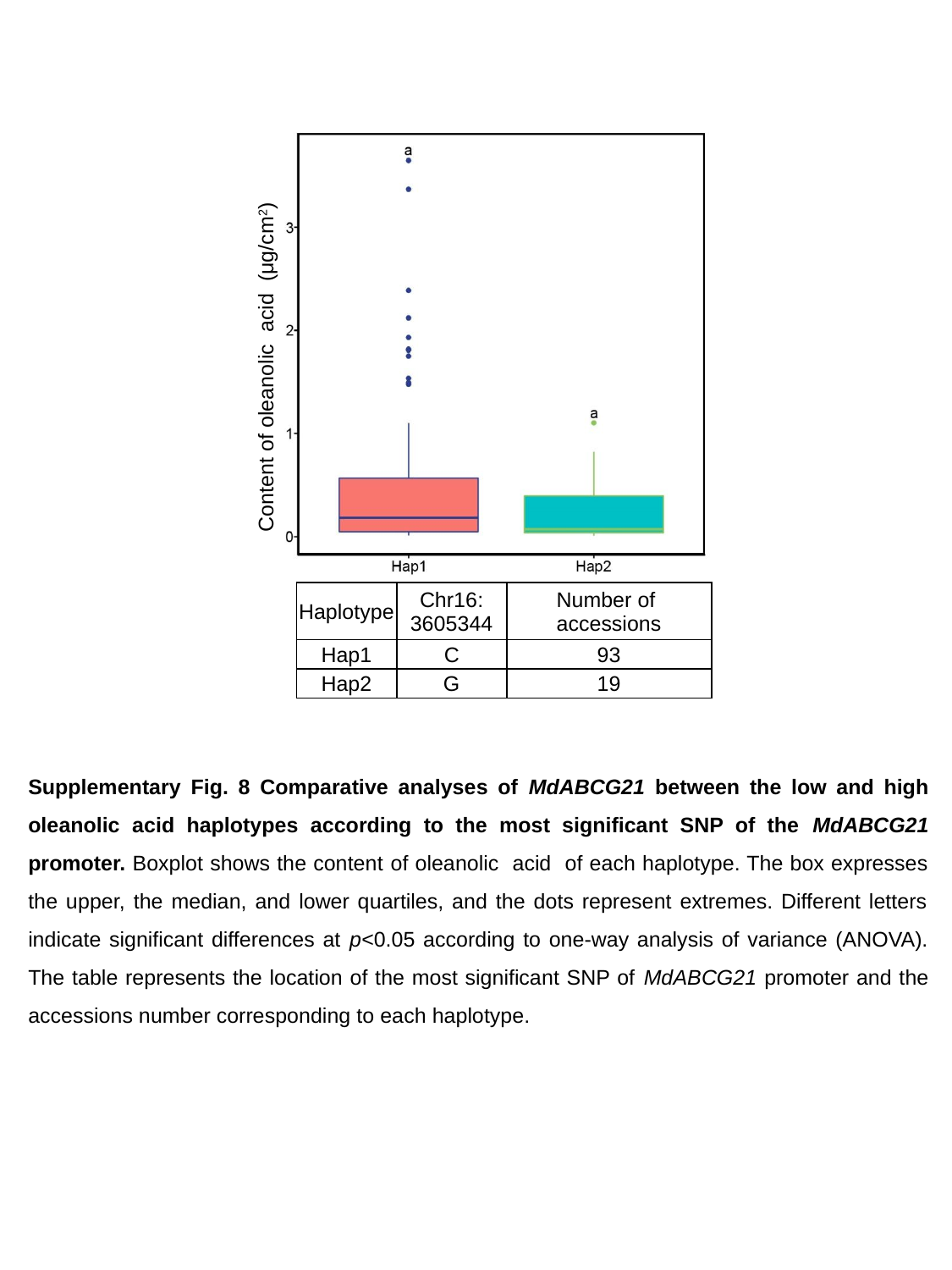

Content of oleanolic acid (μg/cm2)
| Haplotype | Chr16: 3605344 | Number of accessions |
| --- | --- | --- |
| Hap1 | C | 93 |
| Hap2 | G | 19 |
Supplementary Fig. 8 Comparative analyses of MdABCG21 between the low and high oleanolic acid haplotypes according to the most significant SNP of the MdABCG21 promoter. Boxplot shows the content of oleanolic acid of each haplotype. The box expresses the upper, the median, and lower quartiles, and the dots represent extremes. Different letters indicate significant differences at p<0.05 according to one-way analysis of variance (ANOVA). The table represents the location of the most significant SNP of MdABCG21 promoter and the accessions number corresponding to each haplotype.

## Slide 9
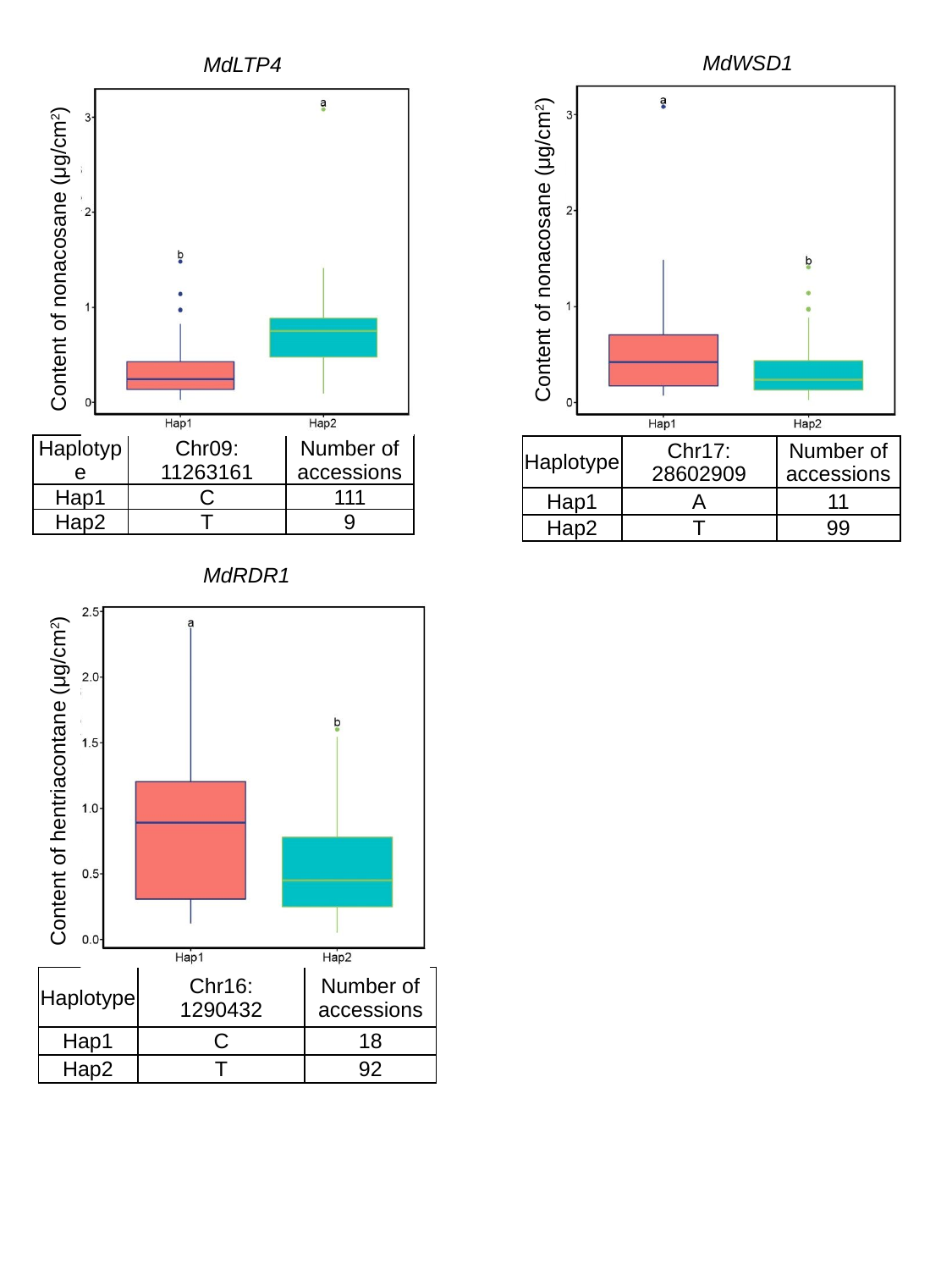

MdWSD1
Content of nonacosane (μg/cm2)
MdLTP4
Content of nonacosane (μg/cm2)
| Haplotype | Chr09: 11263161 | Number of accessions |
| --- | --- | --- |
| Hap1 | C | 111 |
| Hap2 | T | 9 |
| Haplotype | Chr17: 28602909 | Number of accessions |
| --- | --- | --- |
| Hap1 | A | 11 |
| Hap2 | T | 99 |
MdRDR1
Content of hentriacontane (μg/cm2)
| Haplotype | Chr16: 1290432 | Number of accessions |
| --- | --- | --- |
| Hap1 | C | 18 |
| Hap2 | T | 92 |

## Slide 10
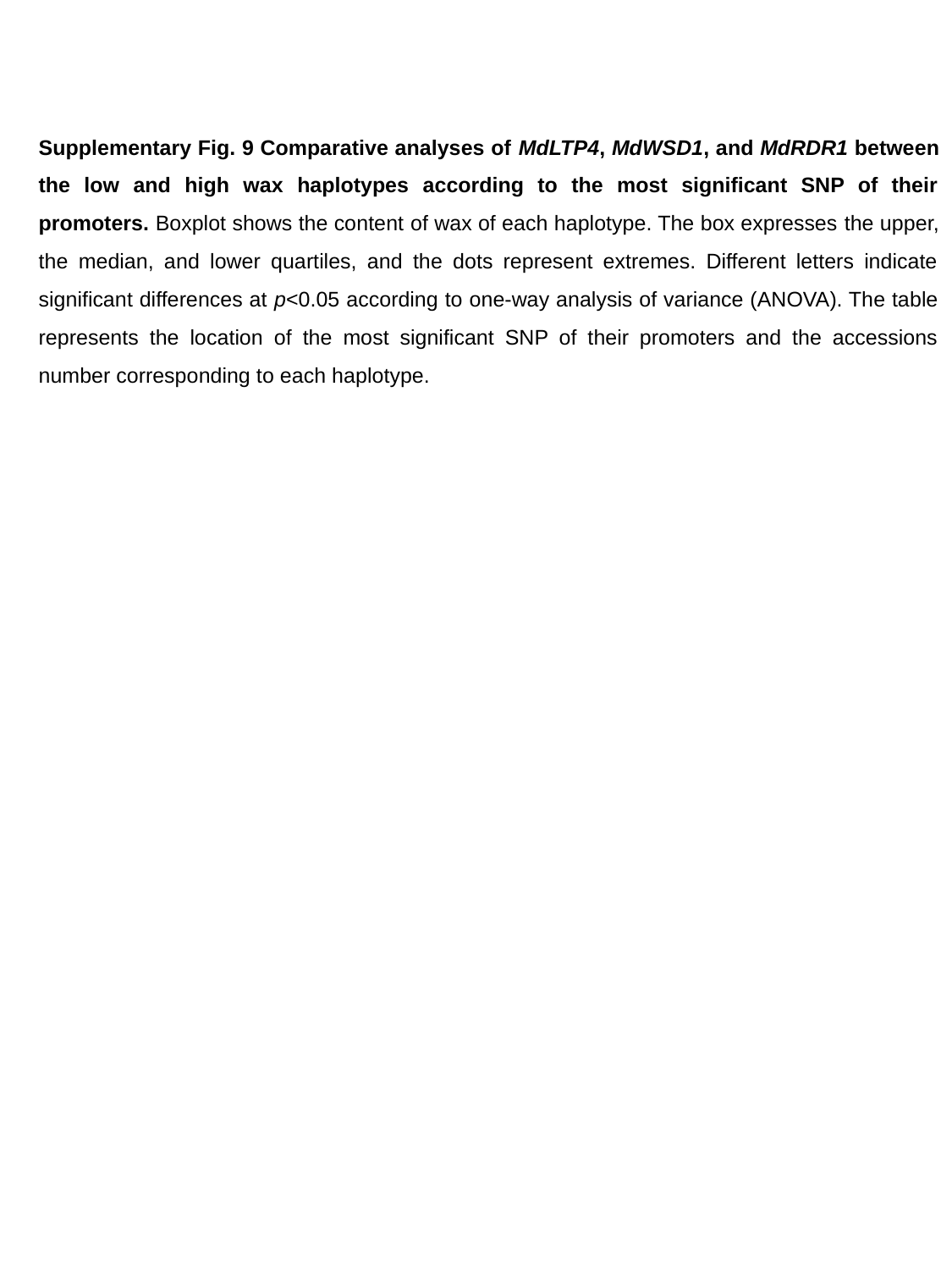

Supplementary Fig. 9 Comparative analyses of MdLTP4, MdWSD1, and MdRDR1 between the low and high wax haplotypes according to the most significant SNP of their promoters. Boxplot shows the content of wax of each haplotype. The box expresses the upper, the median, and lower quartiles, and the dots represent extremes. Different letters indicate significant differences at p<0.05 according to one-way analysis of variance (ANOVA). The table represents the location of the most significant SNP of their promoters and the accessions number corresponding to each haplotype.
